# Maize Seed Germination Under Low-Temperature Stress Impacts Seedling Growth Under Normal Temperature by Modulating Photosynthesis and Antioxidant Metabolism

**DOI:** 10.3389/fpls.2022.843033

**Published:** 2022-03-03

**Authors:** Aiju Meng, Daxing Wen, Chunqing Zhang

**Affiliations:** State Key Laboratory of Crop Biology, Agronomy College, Shandong Agricultural University, Tai’an, China

**Keywords:** seed germination, seedling growth, low-temperature stress, maize, transcriptome

## Abstract

Spring maize is usually subjected to low-temperature stress during seed germination, which retards seedling growth later even under a suitable temperature. However, the mechanism underlying maize seed germination under low-temperature stress impacting seedling growth is still ambiguous. In this study, we used one low-temperature sensitive maize (SM) and one low-temperature resistance maize (RM) to investigate the mechanism. The results showed that the SM line had higher malondialdehyde content and lower total antioxidant capacity (TAC) and germination percentage than the RM line under low-temperature stress, indicating the vulnerability of SM line to low-temperature stress. Further transcriptome analysis revealed that seed germination under low-temperature stress caused the down-regulation of photosynthesis-related gene ontology terms in two lines. Moreover, the SM line displayed down-regulation of ribosome and superoxide dismutase (SOD) related genes, whereas genes involved in SOD and vitamin B6 were up-regulated in the RM line. Kyoto Encyclopedia of Genes and Genomes enrichment analysis revealed that photosynthesis and antioxidant metabolism-related pathways played essential roles in response to low-temperature stress during seed germination. The photosynthetic system displayed a higher degree of damage in the SM line. Both qRT-PCR and physiological characteristics experiments showed similar results with transcriptome data. Taken together, we propose a model for maize seed germination in response to low-temperature stress.

## Introduction

Maize (*Zea mays* L.) originated in tropical and subtropical areas and is naturally sensitive to low-temperature stress, especially during seed germination. Low-temperature limits the spread and production of maize all over the world (Zhang et al., 2020). As spring maize, seed germination and seedling growth at an early stage are usually subjected to low-temperature stress. Despite increased temperature after exposure to low-temperature stress, seedling growth is affected due to the inability of plants to respond quickly to favorable environmental changes (Sowiński et al., 2005). However, the mechanism underlying maize seed germination under low-temperature stress impacting seedling growth is still ambiguous.

Numerous studies have shown that cold stress at the seedling stage affects photosynthesis by reducing the activity of photosystem II (PSII; Savitch et al., 2011). Moreover, cold stress also affects energy collection and preservation at different points, and the reduction of photosynthetic activity varies among different genotypes (Ensminger et al., 2006). The chloroplast ultrastructure of seedlings developed under low-temperature stress is disordered and cannot be repaired after experiencing favorable conditions (Grzybowski et al., 2019). However, the mechanism of low-temperature stress at the germination stage affecting photosynthesis remains unknown.

Low-temperature stress can increase the accumulation of reactive oxygen species (ROS; Li et al., 2019). ROS can cause lipid peroxidation, DNA damage, protein denaturation, carbohydrate oxidation, pigment decomposition, and enzyme activity damage (Bose et al., 2014). Low-temperature tolerance is varied among different genotypes, which may be related to the antioxidant system. ROS scavenging enzymes mainly include superoxide dismutase (SOD), peroxidase (POD), catalase (CAT), glutathione peroxidase (GPX), and ascorbate peroxidase (APX) in plants. Together with antioxidants glutathione and ascorbic acid, these enzymes provide efficient approaches for cells to detoxify $O_{2}^{-}$ and H_2_O_2_ (Romero-Puertas et al., 2006). There is a close relationship between glutathione and cold resistance of maize (Prasad, 1997). Reduced glutathione (GSH), a non-enzymatic antioxidant, is essential for maintaining the redox state of cells (Noctor et al., 2012).

Sugars are essential for plant growth and are involved in response to stress (Zheng et al., 2010). Sucrose is a common substance for energy storage and osmotic regulation in plant cells. Sucrose can also form a sugar layer around the cells, which has a higher membrane phase transition temperature to prevent cell dehydration (Zhang and Bartels, 2018). The increase of sucrose content was the initial reaction of plants exposed to cold conditions (Nägele and Heyer, 2013).

Previous studies have reported the mechanism underlying seedling growth in response to low-temperature stress at the seedling stage (Ma et al., 2015; Zeng et al., 2021). However, spring maize is more susceptible to low-temperature stress at the germination stage than the seedling stage. In this study, two maize inbred lines with different low-temperature resistance were used to investigate the effects of seed germination under low-temperature stress on seedling growth. Maize seed germination at 25°C for 4 days (Normal temperature, NT) was used as control, and maize seed germination at 13°C for 4 days followed by 25°C for 2 days (low-temperature stress followed by normal temperature, LNT) was considered as low-temperature treatment. The physiological experiments showed that the RM line had significantly higher TAC and sucrose content than the SM line under low-temperature stress, partially explaining the different low-temperature resistance phenotypes. Subsequently, we further explore the differences in low-temperature resistance at the transcriptome level. Taken together, the results provide new insights into maize seed germination in response to low-temperature stress.

## Materials and Methods

### Materials

The low-temperature sensitive maize (SM) B283-1 and low-temperature resistance maize (RM) 04Qun0522-1-1maize inbred lines used in this study were bred in our laboratory. They were grown at the experimental station (36°90′N, 117°90′E) of Shandong Agricultural University, Shandong, China. Seeds were sown on 12 June 2019. The plant density was 67,500 plants/ha. Seeds used in this study were harvested 50 days after pollination.

### Evaluation of Seed Germination

Seed germination was evaluated according to a previous study with some modifications (Wen et al., 2018). The bottom of a sprouting bed consisted of 4 cm height silica sand (diameter of 0.05–0.8 mm) with 60% saturation moisture content in a germination box. Randomly selected 30 maize seeds were sown on the surface of the sprouting bed, and then they were covered with 2 cm height silica sand with 60% saturation moisture content. Subsequently, the germination boxes were placed in different germination conditions for various treatments. The germination boxes were placed in a growth chamber at 13°C for 4 days to detect the percentage of seeds showing radicle protrusion. The germination boxes were placed in a growth chamber at 25°C for 7 days or at 13°C for 7 days to measure germination percentage. A seed was considered as germinating seed when the radicle was similar to seed length and the germ was similar with half of the seed length. Moreover, some germination boxes were placed in a growth chamber at 25°C for 4 days (NT) as control, and some germination boxes were placed in a growth chamber at 13°C for 4 days followed by 25°C for 2 days (LNT) as low-temperature treatment. All tissues of the two inbred lines under NT and LNT were used for later experiments. Each treatment included three replicates.

### Measurement of Total Antioxidant Capacity

TAC was measured by a 2,2′-azino-bis (3-ethylbenzothiazoline-6-sulfonic acid) diammonium salt (ABTS) assay kit (Comin, Suzhou, China) following the manufacturer’s protocols. ABTS, a colorless substance, can be oxidized to a stable blue–green cationic radical form ABTS^+^ (Scalzo et al., 2005). The absorption peak of ABTS^+^ is at 734 nm. When the tested substance is added to the ABTS^+^ solution, the antioxidant components can react with ABTS^+^ and fade the reaction system. The change of absorbance at 734 nm was detected. The antioxidant capacity quantified with Trolox was used as a control. The results were expressed in μmol Ttoloox/g fresh weight (FW). Three biological replicates were used for each treatment.

### Measurement of Lipid Peroxidation

The content of thiobarbituric acid (TBA) reactive substances (TBARS) can be used to assess lipid peroxidation (Checchio et al., 2021). In this study, lipid peroxidation was detected using a commercial kit (Comin, Suzhou, China). The results were expressed in nmol/g FW. Three biological replicates were used for each treatment.

### Measurement of Sucrose Content

The determination principle of sucrose content is that alkali is used to heat the sample together to destroy the reducing sugar (Handel, 1968). Then, sucrose was hydrolyzed to glucose and fructose under acidic conditions, and fructose reacted with resorcinol to form a colored substance with a characteristic absorption peak at 480 nm. In this study, sucrose content was detected using a sucrose content determination kit (Comin, Suzhou, China) according to the manufacturer’s protocols. The results were expressed in mg/g FW. Three biological replicates were used for each treatment.

### Measurement of SOD Activity

SOD activity was determined by the photoinhibition of nitro blue tetrazole (NBT) according to a previous study (Giannopolitis and Ries, 1977). In this study, SOD activity was measured using a SOD determination kit (Comin, Suzhou, China) according to the manufacturer’s protocols. The results were expressed in U/g FW. Three biological replicates were used for each treatment.

### Measurement of POD Activity

POD activity was determined by the method of guaiacol oxidation (Zhang et al., 2015a). In this study, POD activity was detected using a POD determination kit (Comin, Suzhou, China) according to the manufacturer’s protocols. The results were expressed in U/kg FW. Three biological replicates were used for each treatment.

### Measurement of Chlorophyll Content

Chlorophyll contents of the two inbred lines germinated at 25°C for 6 days or germinated at 13°C for 4 days followed by 25°C for 4 days were measured by Soil and Plant Analyzer Development (SPAD) 502 chlorophyll meter (Konica Minolta Inc., Japan). Three biological replicates were used for each treatment.

### RNA-seq and Transcriptome Analysis

All tissues of the two inbred lines under NT and LNT conditions were used for RNA extraction. After quick freezing with liquid nitrogen, samples from each replication were pooled and stored at −80°C. Three biological replicates were used for each treatment. Total RNA was extracted using a biospin plant total RNA extraction kit (Bioflux, Beijing, China) according to the manufacturer’s protocols. Libraries were generated using NEBNext^®^ UltraTM RNA Library Prep kit for Illumina^®^ (NEB, United States) following the manufacturer’s protocols. Illumina sequencing was performed as described previously (Yu et al., 2021). Hisat2 (v2.0.5) was used to build an index of the reference genome (B73 v4),^1^ and then paired-end clean reads were aligned to the reference genome (Yu et al., 2021). Differential expression analyses of two groups (three biological replicates per treatment) were performed using the DESeq2 R package (v1.16.1). The resulting values of *p* were adjusted (padj) using Benjamini and Hochberg’s approach for controlling the false discovery rate. Differentially expressed genes (DEGs) with padj <0.05 and |log_2_Fold change| ≥ 1were used for further analyses.

To understand the main biological functions of DEGs in maize, we carried out an enrichment analysis of DEGs. ClusterProfiler R package (v3.4.4) was used to achieve Gene Ontology (GO) enrichment analysis of DEGs (Yu et al., 2012). GO terms with corrected *p* < 0.05 were considered as significantly enriched GO terms. To understand the primary metabolic pathway of DEGs in maize, we carried out the Kyoto Encyclopedia of Genes and Genomes (KEGG) enrichment analysis of DEGs. ClusterProfiler R package (v3.4.4) was used to test the statistical enrichment of DEGs in the KEGG pathway (Yu et al., 2012). Similarly, the KEGG pathways with corrected *p* < 0.05 were assigned as significantly enriched pathways.

### qRT-PCR

To validate the DEGs identified from transcriptome analysis, six genes were randomly selected for a qRT-PCR assay: *Zm00001d002611* (SOD 13), *Zm00001d025103* (Amine oxidase1), *Zm00001d027557* (Glutathione transferase 31), *Zm00001d027422* (PsbP-like protein 1), *Zm00001d021906* (Light harvesting complex A2), and *Zm00001d046170* (phosphoenolpyruvate carboxylase1). Primers were designed using the primer-premier software (v6.0; Supplementary Table S6). The maize *Actin* gene (*Zm00001d010159*) was used as an internal control (Zhang et al., 2015b). An ABI StepOne Plus Real-time PCR System (Applied Biosystems, CA, United States) was used to perform qRT-PCR according to the instructions of SYBR^®^ Green Real-time PCR Master Mix (Takara, Dalian, China). The 2^−ΔΔCT^ method was used to calculate the relative expression of genes (Livak and Schmittgen, 2001).

### Statistical Analysis

Multiple comparisons were performed using Duncan’s test at the 0.05 significance level. All the tests were conducted using SPSS Version 21.0 for Windows (SPSS, Chicago, IL, United States).

## Results

### The SM Line Is More Vulnerable to Low-Temperature Stress

**Figure 1:**
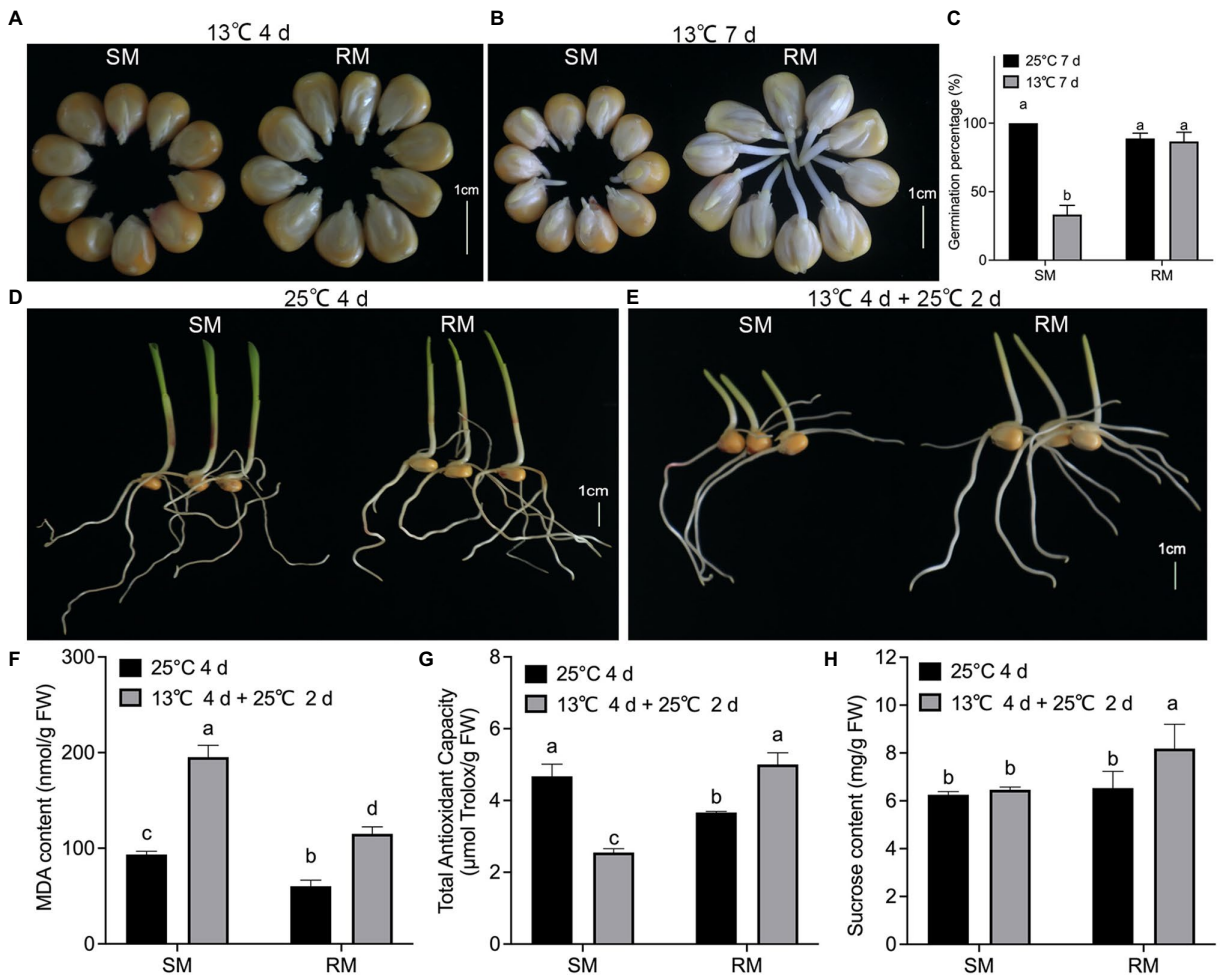
Effects of maize seed germination under low-temperature stress on subsequent seedling growth under normal temperature. **(A)** Phenotypes of seed germination at 13°C for 4 days. **(B)** Phenotypes of seed germination at 13°C for 7 days. **(C)** Germination percentage. **(D)** Phenotypes of seed germination at 25°C for 4 days. **(E)** Phenotypes of seed germination at 13°C for 4 days followed by 25°C for 2 days. Scale bar, 1 cm. **(F)** Malondialdehyde (MDA) content. **(G)** Total antioxidant capacity. **(H)** Sucrose content. Data are means ± SD (*n* = 3 replications of thirty plants). Different letters indicate significant differences among means under different treatments ( *p* < 0.05). FW: fresh weight.

To investigate the effects of maize seed germination under low-temperature stress on subsequent seedling growth under normal temperature, we first measured some traits of two maize inbred lines with different low-temperature resistance. When maize seeds germinated at 13°C for 4 days, the percentage of seeds showing radicle protrusion in the RM line was higher compared to the SM line (Figure 1A). In seed testing, maize seeds germinating at 25°C for 7 days are usually used for evaluating seed germination percentage. The germination percentage of the SM line decreased about 60% at 13°C for 7 days, while there was no significant difference in germination percentage between seeds germinated at 13°C for 7 days and seeds germinated at 25°C for 7 days in the RM line (Figures 1B,C). Maize seeds germinating at 25°C for 4 days (NT) are usually used for evaluating seed germination energy in seed testing. In this study, the two inbred lines had similar germination energy and seedling length (Figure 1D). To investigate the effects of maize seed germination under low-temperature stress impacting seedling growth under normal temperature, we set a similar accumulated temperature between NT and low-temperature stress followed by normal temperature (LNT) treatment. Given the same accumulated temperature, maize seeds first germinated at 13°C for 4 days followed by 25°C for 2 days under LNT treatment. At this time, the RM line had notably higher seedlings than the SM line (Figure 1E). Therefore, these results suggested that the SM line was more vulnerable to low-temperature stress. Subsequently, we detected the changes in lipid peroxidation, expressed as malondialdehyde (MDA) content, TAC, and sucrose content. Although MDA contents of the two lines under LNT were about twice as high as those under NT, the RM line under LNT had a lower MDA content (115 nmol/g FW) than the SM line under LNT (195 nmol/g FW; Figure 1F). TAC decreased in the SM line but increased in the RM line under LNT treatment compared with NT treatment (Figure 1G). TAC under LNT (2.6 μmol Trolox/g FW) decreased to about half of that under NT (5.0 μmol Trolox/g FW) in the SM line. Moreover, the RM line had notably higher sucrose content under LNT than under NT (Figure 1H). Taken together, seeds germinated under low-temperature stress increased TAC and sucrose content in the RM line, which might be related to low-temperature resistance.

### Transcriptome Analysis of Maize Seed Germination in Response to Low-Temperature Stress

**Figure 2:**
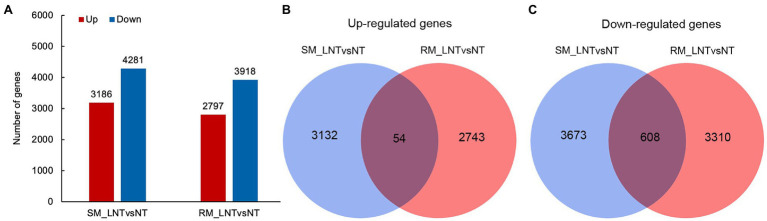
Differentially expressed genes in two maize inbred lines. **(A)** The number of up-regulated and down-regulated differentially expressed genes (DEGs) between low-temperature sensitive maize (SM) and low-temperature resistance maize (RM) lines. **(B)** Venn diagram of up-regulated DEGs in SM_LNTvsNT and RM_LNTvsNT. **(C)** Venn diagram of down-regulated DEGs in SM_LNTvsNT and RM_LNTvsNT. SM: low-temperature sensitive maize inbred line; RM: low-temperature resistant maize inbred line. NT treatment: seeds germinated at 25°C for 4 days. LNT treatment: maize seeds germinated at 13°C for 4 days followed by 25°C for 2 days. SM_LNTvsNT: SM line samples under LNT compared with those under NT. RM_LNTvsNT: RM line samples under LNT compared with those under NT.

To explore genes and metabolic pathways that control maize seed germination in response to low-temperature stress, we selected samples of RM and SM under NT and LNT for transcriptome analysis. After removing adapters and sequences with low-quality regions, there remained approximately 40–50 million clean reads (Supplementary Table S1). Then, about 35–46 million clean reads were mapped to the maize genome. These clean reads included 83–88% uniquely mapped reads and 2.5–3.0% multiple mapped reads. DESeq2 R package was used to identify DEGs using padj <0.05 and |log_2_Fold change| ≥ 1 as the cutoff. The results displayed that 3,186 genes were significantly up-regulated and 4,281 genes were significantly downregulated in the SM line under LNT compared with those under NT (SM_LNTvsNT). Moreover, 2,797 genes were significantly up-regulated and 3,918 genes were significantly downregulated in the RM line under LNT compared with those under NT (RM_LNTvsNT; Figure 2A). Venn diagram showed that common up-regulated genes (54) were fewer than common down-regulated genes (608), most DEGs were specific in SM_LNTvsNT and RM_LNTvsNT (Figures 2B,C).

### Maize Seed Germination Under Low-Temperature Stress Cause Down-Regulation of Photosynthesis-Related Go Terms

**Figure 3:**
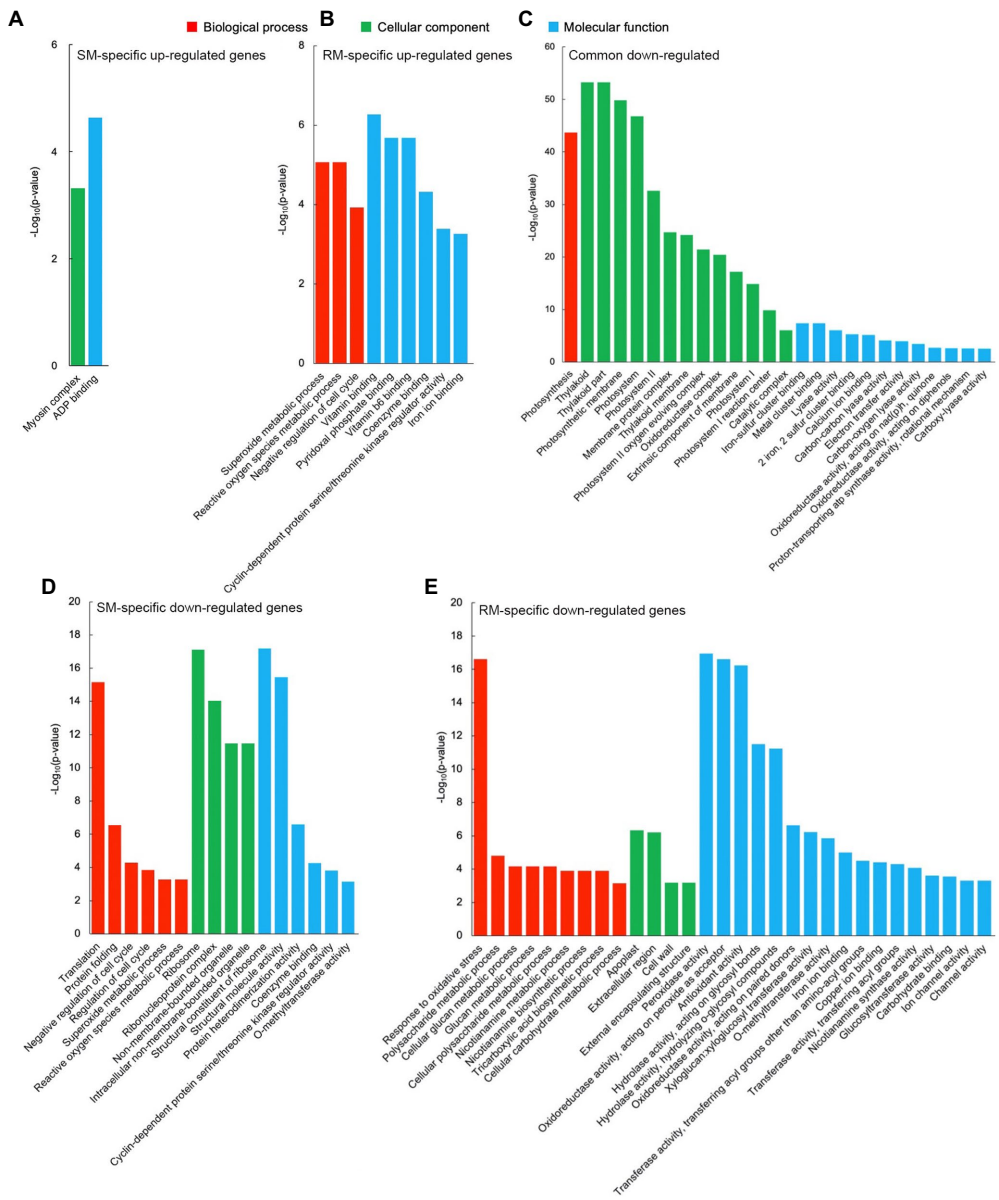
Significantly enriched gene ontology (GO) terms in **(A)** SM-specific up-regulated genes. **(B)** RM-specific up-regulated genes. **(C)** Common down-regulated genes. **(D)** SM-specific down-regulated genes. **(E)** RM-specific down-regulated genes in SM_LNTvsNT and RM_LNTvsNT. SM: low-temperature sensitive maize inbred line; RM: low-temperature resistant maize inbred line. NT treatment: seeds germinated at 25°C for 4 days. LNT treatment: maize seeds germinated at 13°C for 4 days followed by 25°C for 2 days. SM_LNTvsNT: SM line samples under LNT compared with those under NT. RM_LNTvsNT: RM line samples under LNT compared with those under NT.

GO enrichment analysis displayed that there was no significantly enriched GO term for common up-regulated genes, which might be due to the low number of DEGs. For these SM-specific up-regulated DEGs, there were only two significantly enriched GO terms, i.e., myosin complex (GO: 0016459, *p* = 4.82 × 10^−4^) in the cellular component group and ADP binding (GO: 0043531, *p* = 2.32 × 10^−5^) in the molecular function group (Figure 3A). For these RM-specific up-regulated DEGs, there were nine significantly enriched GO terms. Among them, the most significantly enriched GO terms were superoxide metabolic (GO: 0006801, *p* = 8.55 × 10^−6^) in the biological process group, vitamin binding (GO: 0019842, *p* = 5.35 × 10^−7^) in the molecular function group, which might play essential roles in RM resistant to low-temperature stress (Figure 3B).

Compared with the up-regulated GO terms, there were more down-regulated GO terms. For these common down-regulated DEGs, the most significantly enriched GO terms were photosynthesis (GO: 0015979, *p* = 2.16 × 10^−44^) in the biological process group, thylakoid (GO: 0009579, *p* = 5.33 × 10^−54^) in the cellular component group, iron–sulfur cluster binding (GO: 0051536, *p* = 4.38 × 10^−8^) in the molecular function group (Figure 3C). Moreover, many photosynthesis-related GO terms were also enriched. The results indicated that maize seed germination under low-temperature stress caused the down-regulation of photosynthesis-related GO terms in the two inbred lines. Translation (GO: 0006412, *p* = 6.79 × 10^−16^) in the biological process group, ribosome (GO: 0005840, *p* = 7.64 × 10^−18^) in the cellular component group, structural constituent of ribosome (GO: 0003735, *p* = 6.47 × 10^−18^) in the molecular function group, represented the most markedly enriched GO terms in SM-specific down-regulated DEGs, suggesting that ribosomes of SM line might be damaged by low-temperature stress (Figure 3D). For RM-specific down-regulated DEGs, the most prominently enriched GO terms were response to oxidative stress (GO: 0006979, *p* = 2.41 × 10^−17^) in the biological process group, apoplast (GO: 0048046, *p* = 4.58 × 10^−7^) in the cellular component group, peroxidase activity (GO: 0004601, *p* = 1.14 × 10^−17^) in the molecular function group (Figure 3E). The results indicated that peroxidase activity might not be involved in the enhanced antioxidant capacity of the RM line in response to low-temperature stress.

### Photosynthesis and Antioxidant Metabolism Pathways Are Involved in Response to Low-Temperature Stress at the Germination Stage

**Figure 4:**
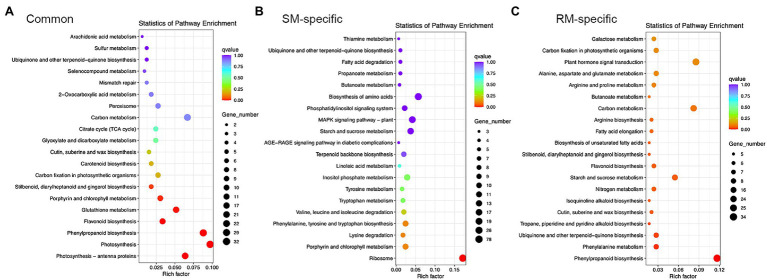
Top 20 KEGG pathways in **(A)** Common DEGs. **(B)** SM-specific DEGs. **(C)** RM-specific DEGs in SM_LNTvsNT, and RM_LNTvsNT. SM: low-temperature sensitive maize inbred line; RM: low-temperature resistant maize inbred line. NT treatment: seeds germinated at 25°C for 4 days. LNT treatment: maize seeds germinated at 13°C for 4 days followed by 25°C for 2 days. SM_LNTvsNT: SM line samples under LNT compared with those under NT. RM_LNTvsNT: RM line samples under LNT compared with those under NT.

KEGG enrichment analysis showed photosynthesis-antenna proteins, photosynthesis, phenylpropanoid biosynthesis, flavonoid biosynthesis, glutathione metabolism, porphyrin and chlorophyll metabolism, stilbenoid, diarylheptanoid, and gingerol biosynthesis were markedly enriched KEGG pathways for common DEGs (Figure 4). Therefore, photosynthesis and antioxidant metabolism pathways might play essential roles in response to low-temperature stress at the germination stage.

Only the ribosome pathway was significantly enriched for SM-specific DEGs, which was also enriched in GO enrichment analysis. In the ribosome pathway, there were 78 DEGs in SM_LNTvsNT (Supplementary Table S2). Of the 78 DEGs, most genes were down-regulated except for three genes (*Zm00001d022111*, *Zm00001d022197*, and *Zm00001d002462*), indicating that low-temperature stress might have a significant influence on the ribosomal pathway of low-temperature sensitive inbred lines. For RM-specific DEGs, the significantly enriched KEGG pathways were related to phenylpropanoid biosynthesis, phenylalanine metabolism, ubiquinone, and other terpenoid-quinone biosynthesis, tropane, piperidine and pyridine alkaloid biosynthesis (Supplementary Table S3). Only one gene was down-regulated among five DEGs involved in tropane, piperidine and pyridine alkaloid biosynthesis.

### The Photosynthetic System of the SM Line Is More Vulnerable to Low-Temperature Stress

**Figure 5:**
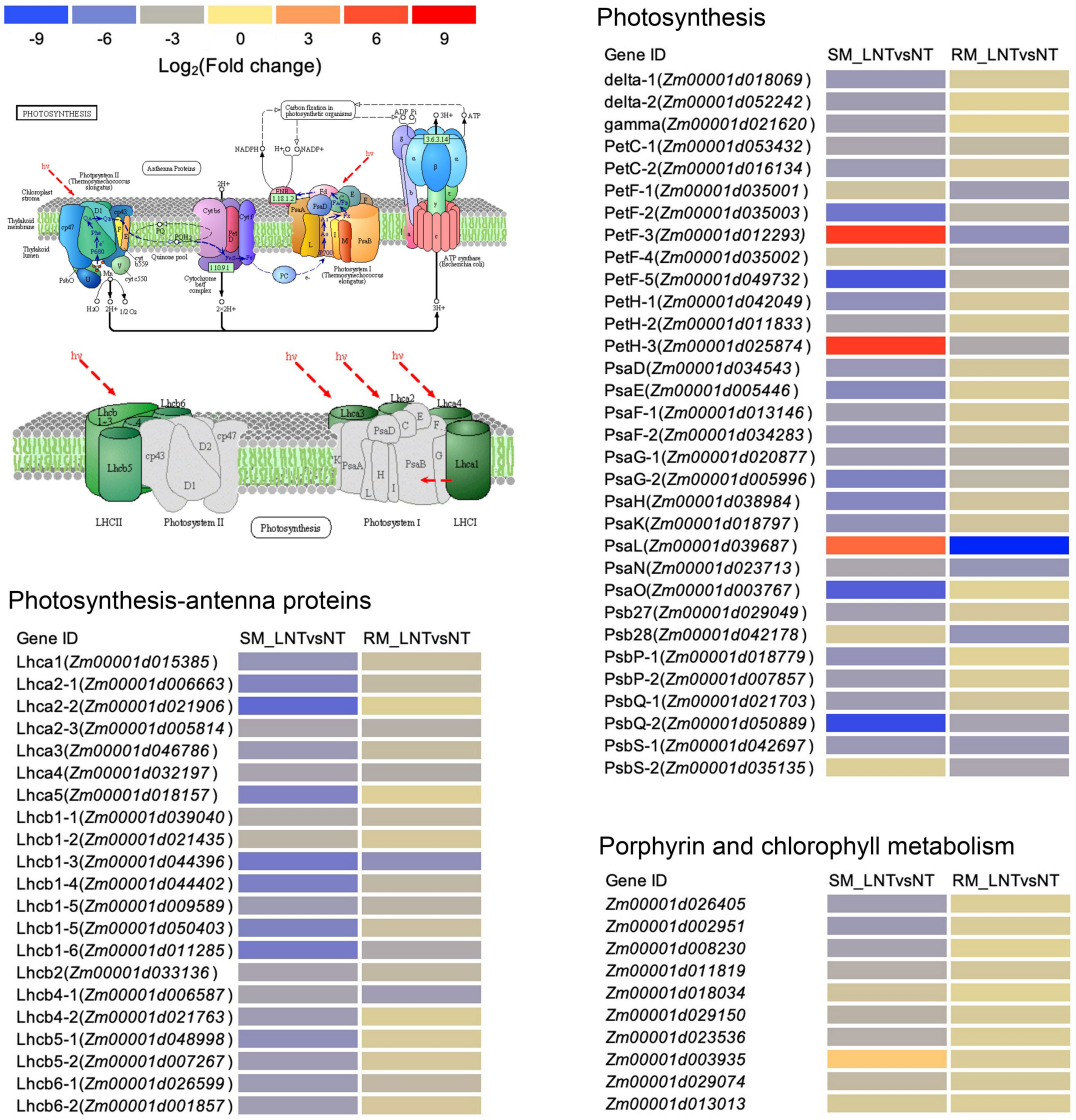
Heat map of photosynthesis-related genes enriched in Kyoto encyclopedia of genes and genomes (KEGG) pathways in SM_LNTvsNT and RM_LNTvsNT. SM: low-temperature sensitive maize inbred line; RM: low-temperature resistant maize inbred line. NT treatment: seeds germinated at 25°C for 4 days. LNT treatment: maize seeds germinated at 13°C for 4 days followed by 25°C for 2 days. SM_LNTvsNT: SM line samples under LNT compared with those under NT. RM_LNTvsNT: RM line samples under LNT compared with those under NT. Detailed lists of the DEGs are shown in Supplementary Table S3. The color code from blue to red suggests the expression level of the DEGs normalized as the log_2_ (Fold change).

The DEGs enriched in photosynthesis-related pathways were mainly located in the chloroplast and annotated to function as antenna proteins, photosystems I and II components, porphyrin, and chlorophyll metabolism-related proteins (Figure 5; Supplementary Table S4). Although DEGs involved in photosynthetic-antenna proteins were all down-regulated in both SM and RM lines, the degree of down-regulation of the DEGs was lower in the RM line than in the SM line. Moreover, most of the DEGs involved in photosynthesis and porphyrin and chlorophyll metabolism pathway were also down-regulated in both SM and RM lines, and the RM line also had a lower degree of down-regulation of the DEGs than the SM line (Figure 5; Supplementary Table S4). Taken together, the photosynthetic system of the SM line was even more damaged when seed germinated under low-temperature stress.

### Validation of Transcriptome Data by qRT-PCR and Physiological Characteristics

**Figure 6:**
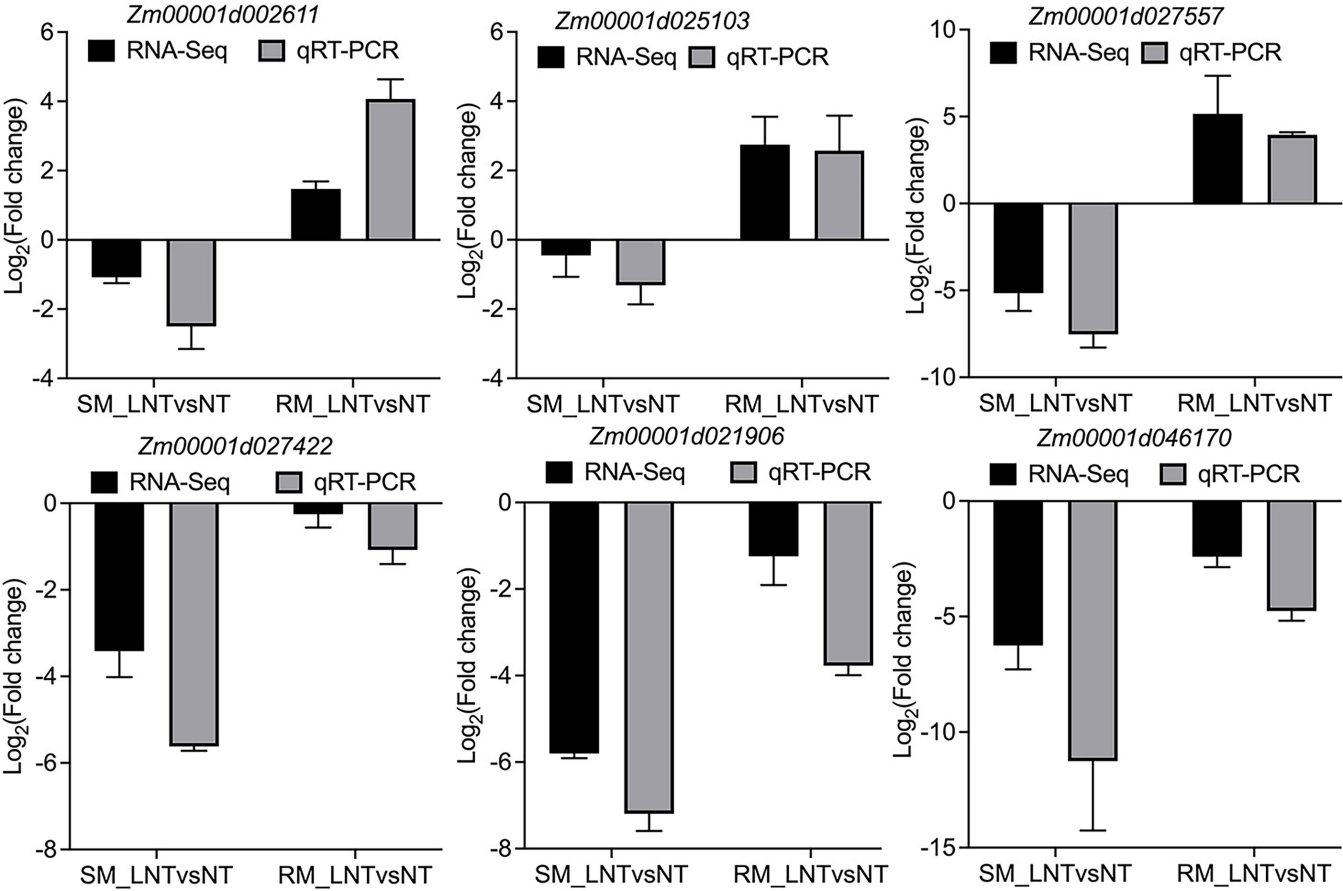
Validation of DEGs by qRT-PCR. *Zm00001d002611* (Superoxide dismutase 13); *Zm00001d025103* (Amine oxidase1); *Zm00001d027557* (Glutathione transferase 31); *Zm00001d027422* (PsbP-like protein 1); *Zm00001d021906* (Light harvesting complex A2); *Zm00001d046170* (phosphoenolpyruvate carboxylase1). The black and gray bars represent the relative expression level from the RNA-seq and qRT-PCR data. The maize *Actin* gene, as an internal control, was used to normalize the expression levels of the target genes. The error bars represent the standard deviations of three replicates. SM: low-temperature sensitive maize inbred line; RM: low-temperature resistant maize inbred line. NT treatment: seeds germinated at 25°C for 4 days. LNT treatment: maize seeds germinated at 13°C for 4 days followed by 25°C for 2 days. SM_LNTvsNT: SM line samples under LNT compared with those under NT. RM_LNTvsNT: RM line samples under LNT compared with those under NT.

**Figure 7:**
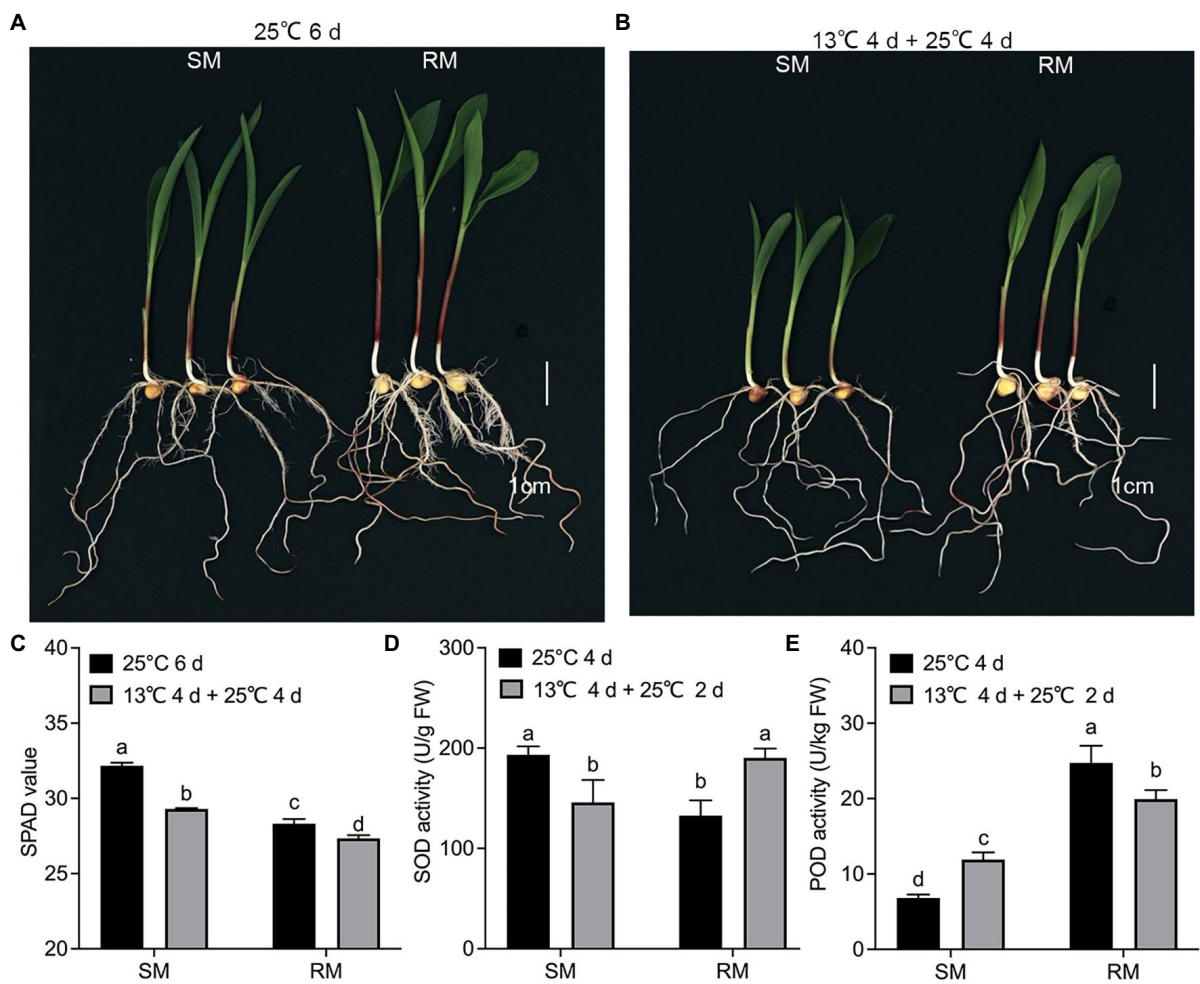
Validation of transcriptome data by physiological characteristics. **(A)** Phenotypes of seed germination at 25°C for 6 days. **(B)** Phenotypes of seed germination at 13°C for 4 days followed by 25°C for 4 days. **(C)** SPAD value. **(D)** Superoxide dismutase. **(E)** Peroxidase. Data are means ± SD (*n* = 3 replications of 30 plants). Different letters indicate significant differences among means under different treatments ( *p* < 0.05). FW: fresh weight. SM: low-temperature sensitive maize inbred line; RM: low-temperature resistant maize inbred line.

All the DEGs showed similar expression patterns in the qRT-PCR assays as their changes of relative expression level identified by RNA-seq, suggesting the transcriptome data were credible (Figure 6). By extending the seedling growth at 25°C for 2 days, we observed that maize seed germination under low-temperature markedly inhibited subsequent seedling growth under normal temperature, especially in the SM line (Figures 7A,B). Subsequently, we further detected the changes in SPAD value, SOD, and POD activities. Both SM and RM lines displayed a significant decrease of SPAD value under LNT, and the level of reduction in the SM line was larger than that in the RM line, which was consistent with the changes of the photosynthetic system from transcriptome analysis (Figure 7C). The SM line had lower SOD activity and higher POD activity under LNT than NT treatment, while the RM line showed the opposite trend of SOD and POD activities, which were consistent with the results of GO enrichment analysis (Figures 7D,E). Moreover, the changes in SOD activities in both SM and RM lines were similar to TAC trends (Figures 1F, 7D). Therefore, SOD activity might play a key role in TAC in seed germination under low-temperature stress.

## Discussion

Low-temperature stress often occurs at the germination stage, which retards the seedling emergence of spring maize. Maize is vulnerable to low-temperature stress at the germination stage and the early stage of seedling establishment (Zhang et al., 2020). Deciphering the mechanism underlying maize seed germination in response to low-temperature stress can help improve low-temperature resistance.

ROS generation in plant cells can be induced by some environmental factors, such as cold (Li et al., 2019), drought (Zheng et al., 2020), heat (Zhao et al., 2018), and cadmium stress (Gu et al., 2019). ROS, as signal molecules, trigger signal transduction pathways in response to these abiotic stresses. In addition, ROS can cause irreversible cell damage through its strong oxidation characteristics, thereby promoting the change of plant morphology and structure and enhancing resistance (Bose et al., 2014; Ohama et al., 2017). ROS can cause lipid peroxidation, DNA damage, protein denaturation, carbohydrate oxidation, pigment decomposition, and enzyme activity damage (Bose et al., 2014). In the present study, both SM and RM lines showed enhanced MDA content in seed germination under low-temperature stress, which was consistent with previous studies.

The detoxification mechanism of ROS plays a vital role in the normal metabolism of plants, especially under stress. The main ROS scavenging enzymes in plants include SOD, POD, CAT, GPX, and APX. Previous studies have shown that CAT and monodehydroascorbate reductase activities are effective screening tools for maize hybrids with cold resistance (Hodges et al., 1997). The activities of antioxidant enzymes significantly increase when maize seeds germinate under low-temperature (Cao et al., 2019). In the present study, the RM line under LNT displayed increased SOD activity and decreased POD activity compared with NT treatment, while the SM line showed opposite trends in both SOD and POD activities (Figures 7D,E). Moreover, GO enrichment analysis showed similar trends with the activities of SOD and POD (Figures 3B,E). Interestingly, the changes in TAC were consistent with SOD activity (Figures 1F, 7D). Therefore, SOD activity might play a key role in the TAC of maize seed germination under low-temperature stress. A previous study has shown that the intrinsic high level of SOD in halophytes is necessary to trigger a series of adaptive responses, and the role of other enzymatic antioxidants might reduce the basic level of H_2_O_2_ (Bose et al., 2014). Whether SOD activity also triggers a series of adaptive responses under low-temperature stress needs further study.

Glutathione metabolism regulates redox-sensitive signal transduction of plant tissues and maintains antioxidant properties (Cnubben et al., 2001; Noctor et al., 2012). Compared with NT treatment, glutathione metabolism-related genes encoding GPX (*Zm00001d026154* and *Zm00001d002704*), glutathione transferase (*Zm00001d018220*, *Zm00001d027557, Zm00001d042102*, *Zm00001d029706* and *Zm00001d043344*), isocitrate dehydrogenase (*Zm00001d044021*) were all up-regulated only in the RM line under LNT (Supplementary Table S5). Of these genes, *Zm00001d026154* and *Zm00001d029706* have been reported to help maize resist drought stress (Zheng et al., 2020). Therefore, *Zm00001d026154* and *Zm00001d029706* might be essential for resistance to various abiotic stresses in maize.

Vitamin B6 contains six forms, pyridoxal, pyridoxamine, pyridoxine, pyridoxal 5′-phosphate (PLP), pyridoxamine 5′-phosphate, and pyridoxine 5′-phosphate, of which PLP is the active form (Denslow et al., 2007). PLP is essential for many biochemical reactions, including decarboxylation, transamination, deamination, racemization, and trans sulfur reactions, which are mainly related to amino acid synthesis (Drewke and Leistner, 2001). Vitamin B6 as a cofactor has been fully confirmed. Moreover, vitamin B6 as an effective antioxidant and a factor that can increase resistance to biotic and abiotic stress has been proved (Huang et al., 2013). Previous studies have shown that VB6 is an effective singlet oxygen quencher, and its quenching rate is equivalent to or higher than that of vitamin C and E, which are known as the two most effective biological antioxidants (Ehrenshaft et al., 1998, 1999; Denslow et al., 2007). In the present study, GO enrichment analysis of RM-specific up-regulated DEGs showed pyridoxal phosphate binding (GO: 0030170, *p* = 2.08 × 10^−6^) and vitamin B6 binding (GO: 0070279, *p* = 3.85 × 10^−8^) were significantly enriched in the molecular function group (Figure 3B). Therefore, vitamin B6 might be involved in enhancing the low-temperature resistance of maize at the germination stage.

Photoinhibition occurs when the harvested light energy exceeds the available energy of chloroplasts or low-temperature sensitive plants exposed to low-temperature stress (Li et al., 2019). Moreover, low-temperature stress can regulate PSII activity, leading to the loss of photosynthetic capacity (Savitch et al., 2011). The down-regulation of light-harvesting complex protein will affect the downstream energy-related processes and ultimately affect plant growth and development (Savitch et al., 2011). Most genes related to photosynthetic apparatus were down-regulated in both RM and SM lines, but the level of reduction of the SM line was greater than that of the RM line (Figure 5). Compared with NT treatment, the RM line showed better recovery ability in photosynthesis than the SM line under LNT. Cold affects photosynthesis through overexcitation of PSII reaction centers and the production of oxygen free radicals (Nie et al., 1992). ROS has harmful effects on photosynthetic devices (Di Fenza et al., 2017). The RM line has higher TAC than the SM line, which might be related to the smaller decrease in photosynthesis in the RM line. After low-temperature treatment, the SPAD values of the two lines decreased significantly, but the level of reduction of the SM line was greater than that of the RM line, which was consistent with the transcriptome data. Therefore, seed germination under low-temperature stress reduced subsequent seedling photosynthesis, but the level of reduction of photosynthesis was different between maize inbred lines with various low-temperature resistance.

Ribosomes are implicated in resistance to various adverse conditions (Garcia-Molina et al., 2020). Many down-regulated DEGs were enriched in the ribosome (GO: 0005840, *p* = 7.64 × 10^−18^) in the SM line (Supplementary Table S2). In mammalian cells, nucleolus, especially ribosome, is considered to be the hub of integrating cell response to adverse conditions (Yang et al., 2018; Pfister, 2019). Some plant species have similar regulatory roles of ribosomes under abiotic and biotic stresses (Garcia-Molina et al., 2020). So far, many ribosomal proteins (RPS) mutants with defects in chloroplast ribosomes have been reported in plants (Schultes et al., 2000; Xu et al., 2013; Zhang et al., 2016). In the present study, gene (*Zm00001d012353*) encoding 30S ribosomal protein S17 chloroplastic was significantly down-regulated in SM_LNTvsNT. The first plant plastid ribosomal protein mutant (high chlorophyll fluorescence 60) in maize displays an unstable light green effect on seedling growth due to the lack of plastid ribosomal small subunit protein 17 (Schultes et al., 2000). The transcription level of RPS is up-regulated after low-temperature acclimation (Garcia-Molina et al., 2020). The up-regulation of RPS under low-temperature stress is considered to maintain the rate of protein synthesis under adverse thermodynamic conditions (Garcia-Molina et al., 2020). The genes encoding 30S ribosomal protein S1 chloroplastic (*Zm00001d038835*), 30S ribosomal protein S10 chloroplastic (*Zm00001d028153*), 30S ribosomal protein S4 chloroplastic (*Zm00001d047186*), 30S ribosomal protein S6 alpha chloroplastic (*Zm00001d034808*), 50S ribosomal protein L1 chloroplastic (*Zm00001d038084*), 50S ribosomal protein L11 chloroplastic (*Zm00001d027421*), 50S ribosomal protein L17 chloroplastic (*Zm00001d012998*), 50S ribosomal protein L21 chloroplastic (*Zm00001d053377*), 50S ribosomal protein L6 chloroplastic (*Zm00001d047462*) were all down-regulated only in the SM line. The role of translation and ribosome in adaptation to abiotic environment changes has been found in a recent analysis of the corresponding *Arabidopsis* mutants (Reiter et al., 2020). In particular, various examples of impaired cold tolerance due to the inactivation of chloroplast proteins involved in translation have been described, including subunits (Wang et al., 2017), biogenesis factors (Reiter et al., 2020) of the plastid ribosome-associated proteins (Pulido et al., 2018), translation initiation or elongation factors (Liu et al., 2010) and RNA-binding proteins (Kupsch et al., 2012). Therefore, down-regulated chloroplastic ribosomal protein-related genes in the SM line under LNT might cause the down-regulation of genes involved in the photosystem, thereby affecting photosynthesis and decreasing SPAD value.

**Figure 8:**
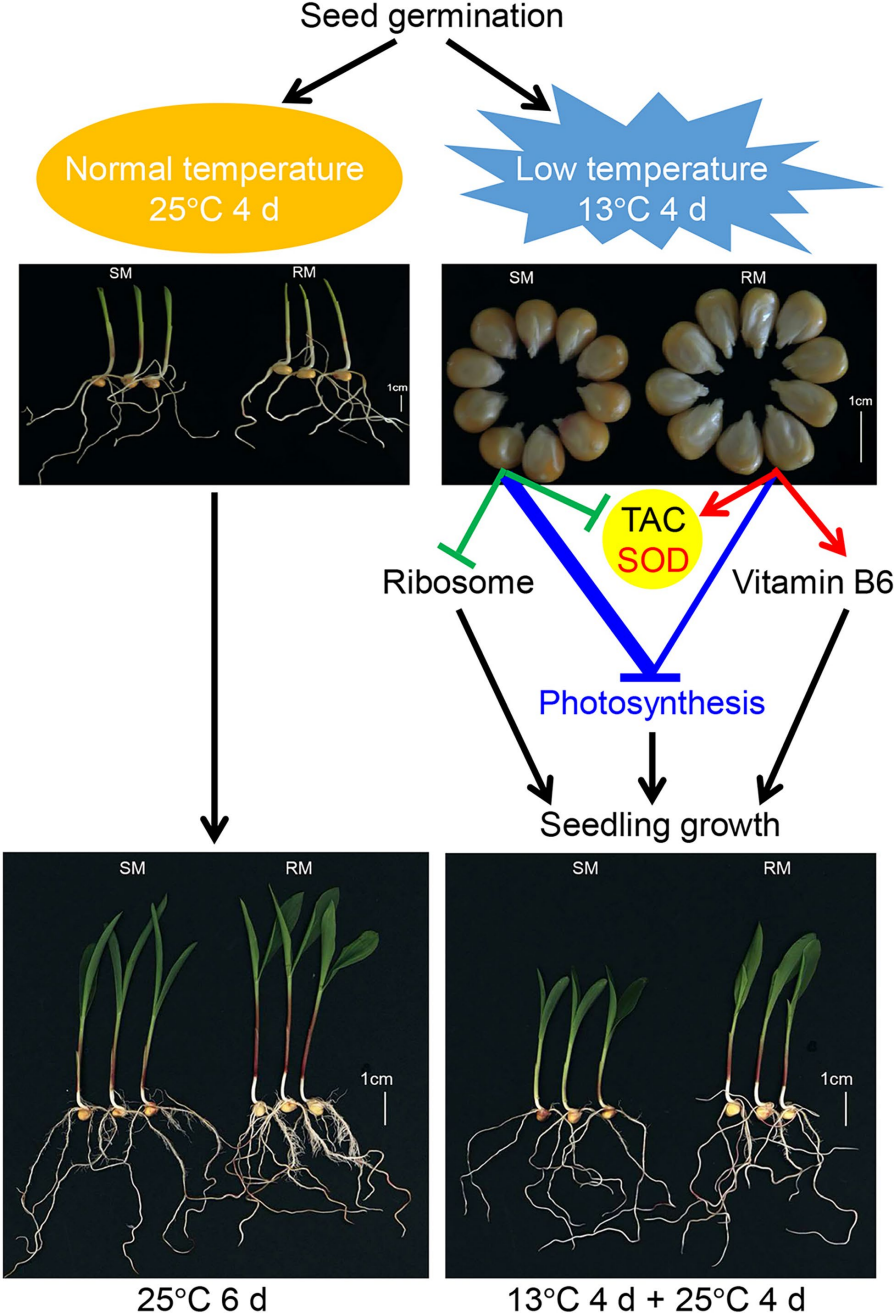
A possible network of maize seed germination under low temperature affecting subsequent seedling growth. The thickened blue line indicates stronger changes induced by low-temperature stress (according to Figure 5).

Overall, we propose a possible network of maize seed germination under low-temperature stress affecting subsequent seedling growth (Figure 8). Maize seed germination under low-temperature stress caused the down-regulation of photosynthesis-related genes in both SM and RM lines, but the degree of down-regulation of the genes was lower in the RM line than in the SM line. Moreover, the SM line displayed the down-regulation of the ribosome and SOD-related genes, whereas genes involved in SOD and vitamin B6 were up-regulated in the RM line. SOD activity might play a key role in the TAC of maize seed germination under low-temperature stress because the changes in TAC were consistent with SOD activity. The inhibition of maize seed germination under low-temperature on seedling growth might be mainly due to impaired photosynthesis. The differences of TAC (especially SOD activity) among various lines might affect low-temperature resistance at the germination stage.

## Conclusion

In summary, maize seed germination under low-temperature stress displayed an increase of lipid peroxidation and inhibited subsequent seedling growth under normal temperature. Transcriptome analysis revealed that photosynthesis and antioxidant metabolism-related pathways played essential roles in seed germination in response to low-temperature stress at the germination stage, and the photosynthetic system of the SM line was more vulnerable to low-temperature stress. Moreover, SOD activity might play a key role in TAC in seed germination under low-temperature stress. Therefore, this study provides new insights into maize seed germination in response to low-temperature stress.

## Data Availability Statement

The original contributions presented in the study are publicly available. This data can be found at: NCBI, BioProject, PRJNA795360.

## Author Contributions

AM, DW, and CZ designed research. AM performed experiments. DW and CZ experimentally guided. AM and DW analyzed the results and wrote the manuscript. All authors contributed to the article and approved the submitted version.

## Funding

This work was supported by the Maize Industry Technology System in Shandong Province (SDAIT-02-02).

## Conflict of Interest

The authors declare that the research was conducted in the absence of any commercial or financial relationships that could be construed as a potential conflict of interest.

## Publisher’s Note

All claims expressed in this article are solely those of the authors and do not necessarily represent those of their affiliated organizations, or those of the publisher, the editors and the reviewers. Any product that may be evaluated in this article, or claim that may be made by its manufacturer, is not guaranteed or endorsed by the publisher.

## Footnotes

^1^ http://www.maizesequence.org/index.html

## Abbreviations

SM: Low-temperature sensitive maize
RM: Low-temperature resistance maize
TAC: Total antioxidant capacity
SOD: Superoxide dismutase
PSII: Photosystem II
ROS: Reactive oxygen species
POD: Peroxidase
CAT: Catalase
GPX: Glutathione peroxidase
APX: Ascorbate peroxidase
GSH: Glutathione
NT: 25°C for 4 days
LNT: 13°C for 4 days and then 25°C for 2 days
ABTS: 2,2′-Azino-bis (3-ethylbenzothiazoline-6-sulfonic acid) diammonium salt
FW: Fresh weight
SPAD: Soil and plant analyzer development
DEGs: Differentially expressed genes.
